# Identifying Biologically Meaningful Hot-Weather Events Using Threshold Temperatures That Affect Life-History

**DOI:** 10.1371/journal.pone.0082492

**Published:** 2013-12-04

**Authors:** Susan J. Cunningham, Andries C. Kruger, Mthobisi P. Nxumalo, Philip A. R. Hockey

**Affiliations:** 1 Percy FitzPatrick Institute, DST/NRF Centre of Excellence, University of Cape Town, Rondebosch, South Africa; 2 Climate Service, South African Weather Service, Pretoria, South Africa; USDA-Agricultural Research Service, United States of America

## Abstract

Increases in the frequency, duration and intensity of heat waves are frequently evoked in climate change predictions. However, there is no universal definition of a heat wave. Recent, intense hot weather events have caused mass mortalities of birds, bats and even humans, making the definition and prediction of heat wave events that have the potential to impact populations of different species an urgent priority. One possible technique for defining biologically meaningful heat waves is to use threshold temperatures (T_thresh_) above which known fitness costs are incurred by species of interest. We set out to test the utility of this technique using T_thresh_ values that, when exceeded, affect aspects of the fitness of two focal southern African bird species: the southern pied babbler *Turdiodes bicolor* (T_thresh_ = 35.5°C) and the common fiscal *Lanius collaris* (T_thresh_ = 33°C). We used these T_thresh_ values to analyse trends in the frequency, duration and intensity of heat waves of magnitude relevant to the focal species, as well as the annual number of hot days (maximum air temperature > T_thresh_), in north-western South Africa between 1961 and 2010. Using this technique, we were able to show that, while all heat wave indices increased during the study period, most rapid increases for both species were in the annual number of hot days and in the maximum intensity (and therefore intensity variance) of biologically meaningful heat waves. Importantly, we also showed that warming trends were not uniform across the study area and that geographical patterns in warming allowed both areas of high risk and potential climate refugia to be identified. We discuss the implications of the trends we found for our focal species, and the utility of the T_thresh_ technique as a conservation tool.

## Introduction

Although increasingly severe heat waves are an almost universal prediction of climate-change models [1–3], there is no universal definition of a heat wave [1]. However, intense hot-weather events have already led to mass breeding failures of birds [4] and mass mortalities of birds [5,6], bats [7] and humans [8], placing an immediate onus on biologists to define and predict significant heat wave events that have the potential to impact local or regional populations of different species. 

For their analyses, Meehl and Tebaldi [1] defined heat waves using criteria based on societal impacts (human health and economies). In other words, they used combinations of climatic conditions that defined some threshold temperature (and its duration) beyond which there were measurable impacts on human wellbeing. From a biological perspective, a critical threshold temperature (T_thresh_) could be defined as one above (or below) which some life-history parameter (e.g. survival or reproduction rate) is negatively impacted in a measurable way, even if the short-term effect is sub-lethal (cf 9). For example, a recent study of southern pied babblers *Turdoides bicolor* in southern Africa’s Kalahari region identified a daily maximum temperature (T_max_) of 35.5°C as a T_thresh_ for these birds: when T_max_ > 35.5°C, the birds were unable to maintain body condition on a day-to-day basis [10]. This does not result in any detectable, short-term mortality, but presumably carries a progressively increasing fitness risk as the duration of the event increases. 

Models of species’ responses to climate change are increasingly incorporating biological processes [11,12], but the links between environmental variation and fitness remain elusive [13] (however, see 14). We suggest that reasons for this include a) a failure to identify relevant T_thresh_ values, and b) the fact that these values will differ between species such that the definition of heat waves becomes species-specific. Furthermore, a number of thermal thresholds may exist within single species, each affecting a different correlate of fitness. For example, a recent study of common fiscals (*Lanius collaris*) identified three temperature thresholds regarding aspects of chick development at the time of fledging (temperatures above 33°C reduced fledgling body mass, above 37°C reduced tarsus length, and above 35°C delayed fledging date; [15]). Despite this, the T_thresh_ approach can still be used to describe heat waves likely to compromise fitness in any given species, either by choosing the lowest known T_thresh_ for that species, the one with the steepest rate of decline in fitness above the threshold, or one related to a life-history stage known to be of particular importance to population dynamics of the species in question. Even when only one candidate T_thresh_ value has been identified for a species of interest, so long as the consequences of exceeding this threshold are understood it will be possible to define hot-weather events in biologically meaningful terms. In the absence of any such analysis, it is impossible to assess whether predicted changes in the frequency or nature of hot-weather events will (or will not) have biological significance. 

We explore changes in the frequency, duration and intensity of hot-weather events in the Kalahari and surrounding regions of South Africa since 1961. This region warmed significantly over the period 1961-2009 [16,17] and is predicted to experience further temperature increases in the coming decades [2,3,18]. These changes are analysed with respect to the lowest known T_thresh_ values for two bird species occurring in the region, drawn from the studies mentioned above – southern pied babblers [10] and common fiscals *Lanius collaris* ([15]).

The first aim of this study is to empiricise those heat wave parameters (defined in terms of T_thresh_ values) that are persistently evoked in climate-change predictions, *viz* frequency, duration and intensity. 

Specifically, we set out to answer the following questions:

1. Has there been a change in the frequency (i.e. the annual total number) of days that reach or exceed southern pied babbler and common fiscal minimum T_thresh_? This trend has been shown very clearly for desert weather stations in Australia, using 40°C as T_thresh_ [19]. *H_0_ = there has been no change in the frequency of ‘hot’ days over time.*
2. Has there been a change in heat waves of differing duration? *H_0_ = heat wave duration has not changed over time.*
3. Have non-heat wave periods (i.e. numbers of successive days when T_max_ did not reach or exceed T_thresh_) changed? *H_0_ = there has been no change in the length of intervals between heat waves (i.e. ‘recovery times’ have remained unchanged).*
4. Has heat wave intensity (see methods) changed? *H_0_ = there has been no change over time in the intensity of heat waves*.

The second aim of the study is to attempt to identify which of these changes are the most significant for the two focal bird species and whether the climate trends differ across the southern Kalahari sector of their ranges. This analysis will provide motivation and rationale for the data that field biologists should be collecting. If, for example, intensity is increasing much faster than frequency (which may vary with T_thresh_), then it is heat wave intensity that should form the primary focus for data collection – not necessarily because intensity will have the greatest biological impact on the species in question, but because, given that it is increasing with greatest rapidity, it is the parameter for which the need to investigate biological impact is most urgent. Furthermore, by examining the strength of climate trends relevant to species of interest across their geographical ranges, areas of greatest vulnerability and even potential climate refugia, may be identified. Importantly, the methodology developed here is not only germane to southern pied babblers and common fiscals but can be replicated for any species, anywhere, providing a) a time series of daily T_max_ readings exists, and b) T_thresh_ values have been determined for the target species.

## Materials and Methods

### Defining T_*thresh*_ values

In this study we use maximum daily air temperature (T_max_), measured by weather stations, as our standard for defining heat waves, for the following reasons

1. T_max_ can be used as a proxy for biologically meaningful temperatures, as it will correlate with the range of operative environmental temperatures (T_e_) available to an animal on any given day (for examples see 10,14,15).
2. T_max_ is collected by weather stations globally and used in climate change models.

**Figure 1:**
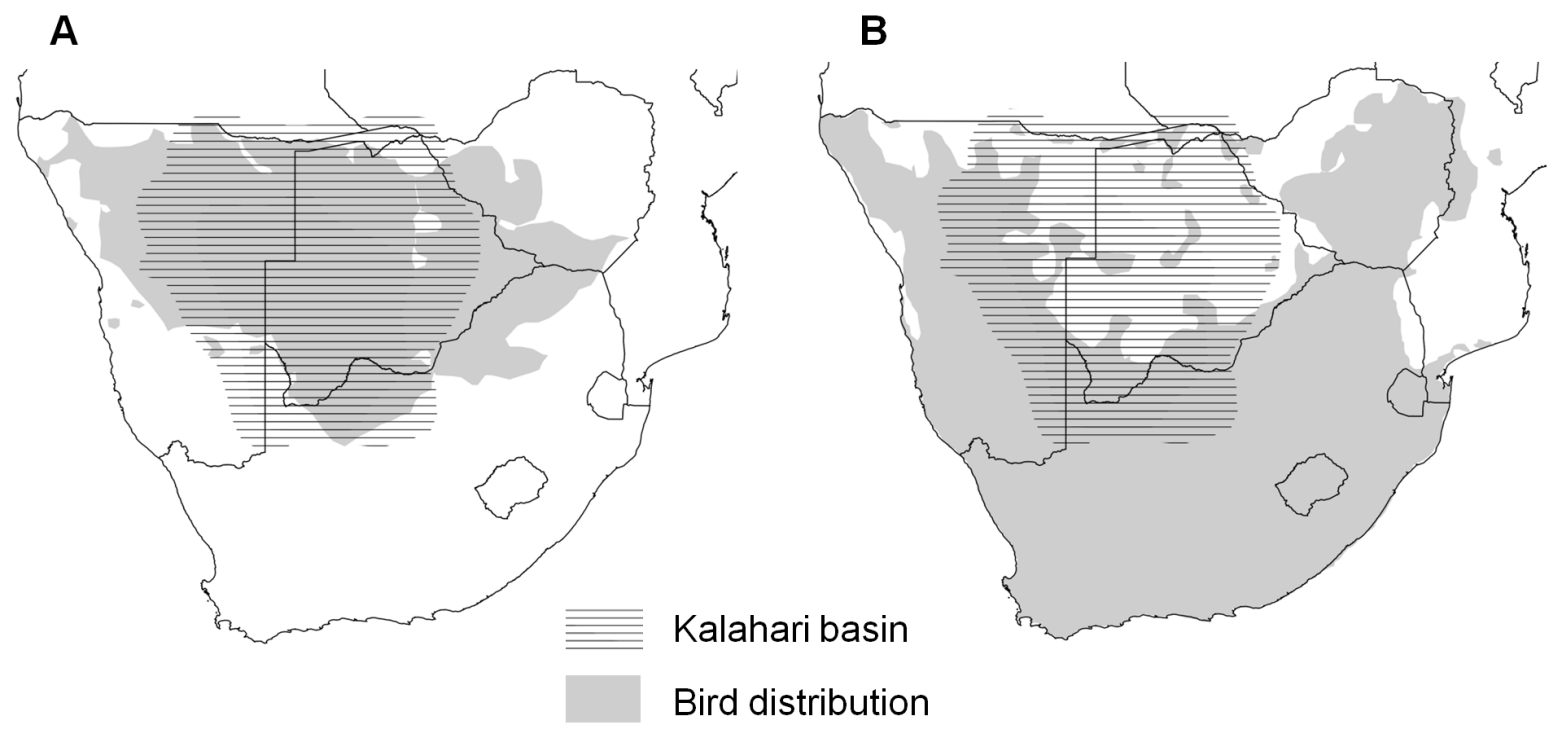
Range maps of focal species in relation to the Kalahari. Southern African distribution of A: southern pied babblers and B: common fiscals, in relation to the Kalahari basin. Range maps drawn from South African Bird Atlas Project data [29].

We define “T_thresh_” as a critical T_max_ threshold above which a species incurs measurable fitness costs. In this study we use T_thresh_ values for two southern Kalahari species drawn from the literature; southern pied babblers [10] and common fiscals (also known as fiscal shrikes[15]). Southern pied babblers (hereafter babblers) are arid-zone specialists with a Southern African range centered on the Kalahari Basin, whereas common fiscals (hereafter fiscals) are a widespread species which, though common in the periphery of the Kalahari Basin, tends to be absent from its centre [20] (Figure 1). T_thresh_ values and their biological relevance to the species in question are presented in Table 1. The studies from which our T_thresh_ values were drawn were each carried out at sites within the South African portion of the southern Kalahari (at Kuruman River Reserve: 26°58’S, 21°49’E; and Tswalu Kalahari Reserve: 27°13'S, 22°22'E).

**Table 1:** T_thresh_ values for focal species and their biological relevance.

Species	T_thresh_	Biological relevance	Potential fitness cost	Reference
Southern pied babbler *Turdoides bicolor*	35.5°C	On days with T_max_ > T_thresh_, babblers may struggle to regain body mass lost the over the previous night (i.e. net 24 hour mass change becomes negative).	Cumulative mass loss over consecutive days > 35.5°C could threaten survival and reproductive success.	du Plessis et al. [10]
Common fiscal *Lanius collaris*	33°C	Body mass at fledge is significantly negatively affected by the number of days during the nestling period on which T_max_ > T_thresh_. The strength of this relationship increases with increasing disparity between T_max_ and T_thresh_.	In passerines, smaller fledglings often have reduced overwinter survival and reduced reproductive success as adults [26,35].	Cunningham et al. [15]

### Temperature data

**Figure 2:**
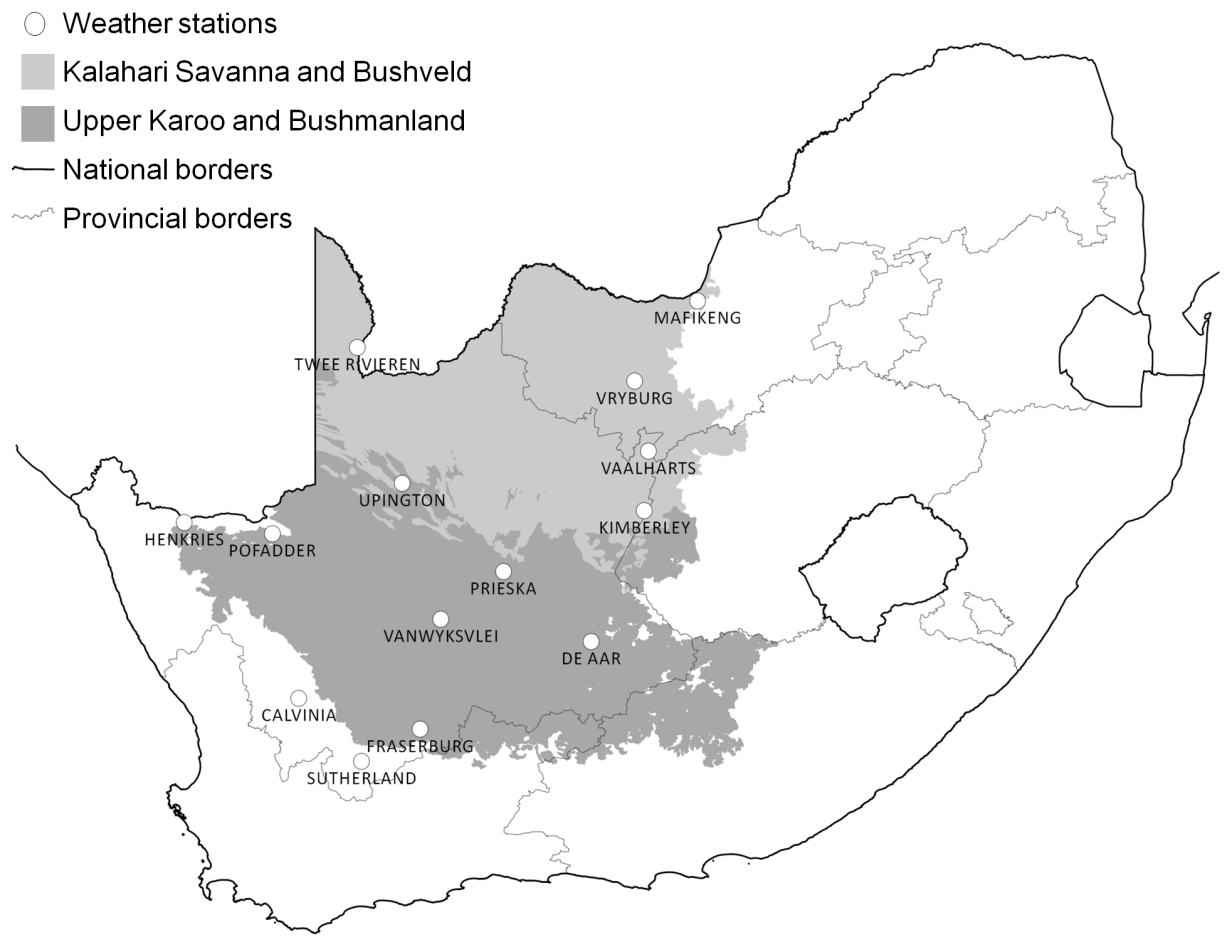
Weather station locations. Locations of weather stations used in this study, in relation to Kalahari and Upper Karoo/Bushmanland biotypes [36].

We selected a common analysis period of 1961–2010 of T_max_ data from the SAWS (South African Weather Service) climate database, in order to obtain the longest possible period with a reasonable number of available climate stations to cover the interior of the Northern Cape and western part of North-West province in South Africa (comprising the South African extent of the Kalahari bioregion and surrounding areas (the Bushmanland and Upper Karoo bioregions; Figure 2).

The data sets of the selected stations were subjected to quality control to remove any values which were possibly erroneous, however this process excluded the colder months when it was highly unlikely that T_max_ greater than either shrike or babbler T_thresh_ would occur. The validity of anomalously high values were checked against the reports of the prevailing weather conditions around the time of observation, and also compared to the values measured at available stations close-by. Consequently, any suspicious data were removed from the time series.

After quality control of the data, the completeness of the time series was verified to comprise more than 80% of the years under consideration. For a particular year to be included, individual months had to have more than 90% of days of data. However, this provision did not apply to the colder winter, late autumn and early spring months.

A total of 14 weather stations were finally accepted for analysis. The SAWS climate number, station name, location in terms of latitude and longitude and altitude are presented for each station in Table 2. Weather station locations are plotted in Figure 2.

**Table 2:** Climate stations utilised in the analysis, listed according to SAWS climate number, with station name, location in terms of latitude and longitude, and altitude.

Number	Name	Latitude (°S)	Longitude (°E)	Altitude (m)
0088293	Sutherland	32.399	20.662	1450
0113025	Fraserburg	31.915	21.507	1266
0134479	Calvinia	31.482	19.762	975
0170009	De Aar	30.674	23.999	1287
0193561	Vanwyksvlei	30.350	21.824	962
0224430	Prieska	29.670	22.740	947
0247668	Pofadder	29.123	19.389	989
0277178	Henkries	28.976	18.094	425
0290468	Kimberley	28.800	24.770	1197
0317475	Upington	28.413	21.259	841
0360597	Vaalharts	27.956	24.844	1175
0461208	Twee Rivieren	26.471	20.610	879
0432237	Vryburg	26.950	24.630	1234
0508047	Mafikeng	25.808	25.543	1282

### Heat wave trend analyses

The T_thresh_ values determined for babblers (T_thresh_ = 35.5°C) and fiscals (T_thresh_ = 33°C) were used as the threshold T_max_ above which heat wave conditions were considered to be reached. As we were interested in trends in the hottest days and periods of consecutive days during each year, we centred our investigations on the austral summer. Thus for the purposes of this work, we defined a ‘year’ as beginning on the 1^st^ July of one calendar year and ending on the 30^th^ June the following calendar year. 

Trends in five ‘heat wave indices’ were analysed for each T_thresh_:

1. Frequency of days per year on which T_max_ ≥ T_thresh_ (units: days);
2. Total number of heat waves per year (heat wave frequency; a heat wave was defined as one or more consecutive days on which T_max_ ≥ T_thresh_);
3. Maximum duration of heat waves, defined as the maximum number of consecutive days per year on which T_max_ ≥ Tthresh; units: days. (We did not analyse trends in average duration because heat wave duration has a systematically non-normal distribution, with short heat waves in early summer followed by longer heat waves in the peak of the season. The additional short heat waves early on in a longer hot season will cancel out the effects of longer heat waves mid-season, making the average duration an uninformative measure);
4. Average duration of intervals between heat waves, defined as the mean number of consecutive days on which T_max_ < T_thresh_ and calculated taking into consideration only the periods between the first and last heat waves of the year (units: days) ; and
5. Intensity of heat waves, calculated as the sum of the daily differences, in degrees Celsius, between T_max_ and T_thresh_ for all the days in the heat wave:

$$\sum_{i=1}^{n}{\left(T{\text{max}}_{i}-\text{Tthresh}\right)}^{}$$

where *n* = total number of days in the heat wave and by definition, all T_max_ ≥ T_thresh_. Average intensity of heat waves in a year was calculated by taking the mean intensity of all heat waves recorded for that year; maximum heat wave intensity was the highest intensity value for a heat wave calculated for that year (units: degree days). 

Trend analyses of the indices were based on linear regression, with significance tested with Student's *t*-test at the 5% level. Once obtained, trend estimates (slope of the regression line) were multiplied by 10 to indicate the trend per decade, which is common practice in climatological trend analysis.

Decadal trend estimates were averaged across all weather stations for each heat wave index, then plotted along with their 95% confidence intervals (CIs) in order to assess which indices showed the strongest trends (steepest rates of increase) across the region. Trends for each weather station were plotted on a map of South Africa using QGIS 1.8 allowing visualisation of spatial patterns.

## Results

### Overall trends

**Figure 3:**
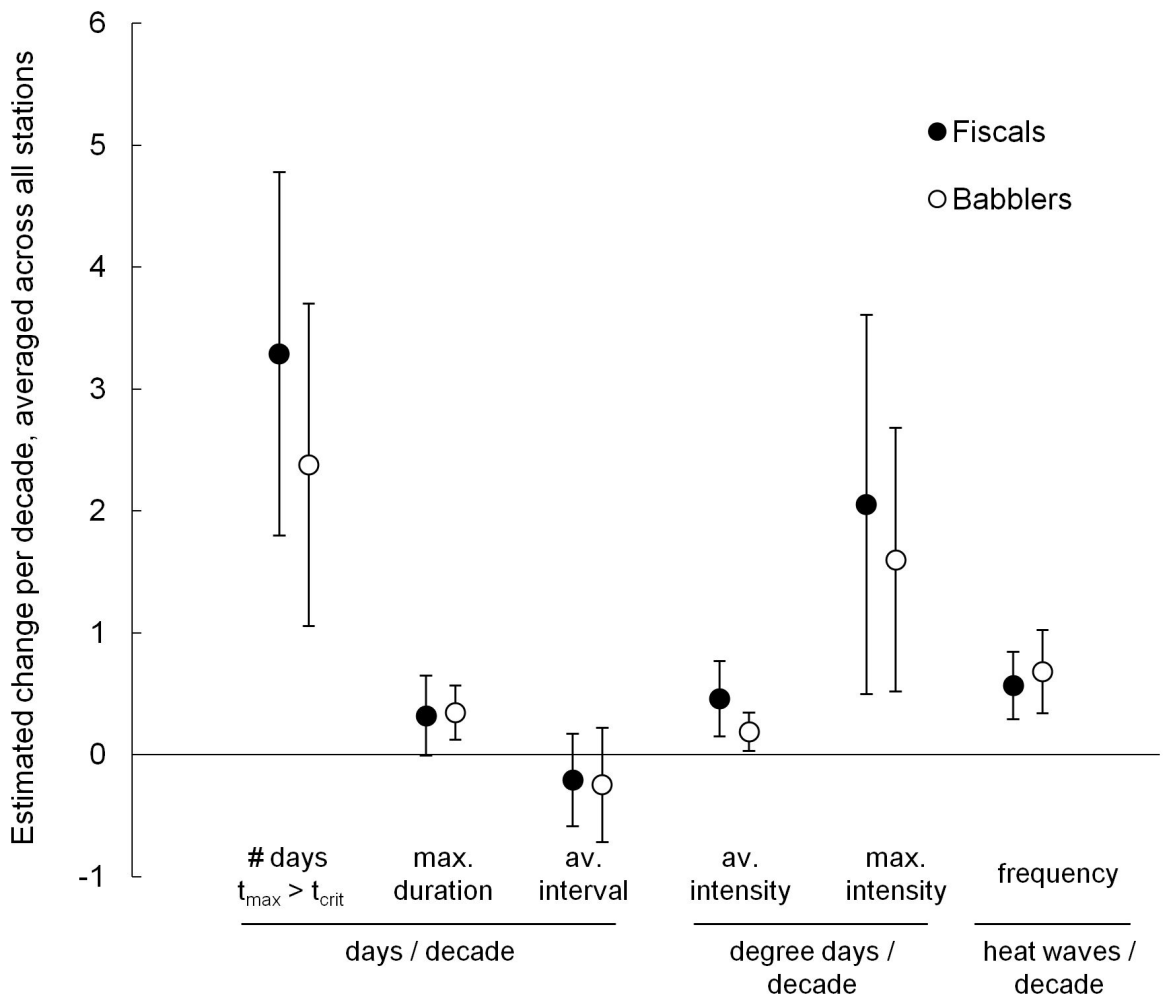
Decadal change in heat wave indices, averaged across weather stations. Effect sizes (averaged slope estimates from trend analyses) and 95% confidence intervals for decadal change in six heat wave indices, averaged across 14 weather stations in the southern Kalahari. Units for the effect sizes vary between the heat wave indices and are indicated on the x-axis to aid comparison of like with like estimates. Heat waves were defined using T_thresh_ values relevant to common fiscals (black circles) and southern pied babblers (white circles).

We found positive mean trends for all heat wave indices (both T_thresh_ values), except for the duration of intervals between heat waves (Figure 3). There was very little difference in the magnitude of the trends for heat waves defined for fiscals versus babblers. Strongest trends for both species were in the number of days per year on which T_max_ > T_thresh_, and in maximum heat wave intensity. Trends for increases in frequency and average intensity of heat waves were also statistically significant (95% CIs did not contain zero), but had smaller estimates (i.e. slower rates of increase). The faster rate of increase in maximum than in average heat wave intensity suggests that while heat waves were becoming more intense on average between 1961 and 2010, variance in intensity was also increasing. Maximum heat wave duration showed a significant (babblers) or near significant (fiscals) positive trend for both species, although estimate sizes were small (<0.5 days/decade increase in duration; Figure 3). 

The duration of intervals between heat waves (the number of consecutive days on which T_max_ < T_thresh_) showed a negative mean trend across all the weather stations, for both species, with 21.4% of stations recording a significant reduction in interval duration for each species (Tables 3 and 4). However, the average effect size was small and confidence intervals contained zero, meaning there was uncertainty as to the overall direction of the trend (Figure 3). 

**Table 3:** Trend estimates for heat waves relevant to common fiscals (T_thresh_ = 33).

Weather station	# days > T_thresh_ (days/decade)	Heat wave frequency (days/decade)	Max. duration of heat waves (days/decade)	Av. intensity of heat waves (degree days/decade)	Max. intensity of heat waves (degree days/decade)	Av. interval between heat waves (days/decade)
Calvinia	3.38 *	1.18 *	0.68 *	0.17	2.17 *	-0.14
De Aar	0.61	0.04	0.46	-0.13	-0.42	-0.09
Fraserburg	3.55 *	1.27 *	0.37	0.35 *	1.33	-0.45
Henkries	2.50	0.02	-0.23	0.62	3.24	-0.11
Kimberley	3.53	0.53	0.69	0.52	3.06	0.10
Pofadder	5.77 *	1.18 *	1.05 *	0.48 *	4.39 *	-0.20
Prieska	3.05	-0.14	-0.98	0.76	-3.32	-0.09
Sutherland	1.12 *	0.68 *	0.24 *	0.15	0.59 *	-2.49 *
Twee Rivieren	6.43 *	-0.14	0.80	1.91 *	6.87	-0.03
Upington	7.84 *	0.60	0.11	1.06 *	2.91	-0.14 *
Vaalharts	2.63	0.87	-0.09	0.13	1.01	-0.18
Vanwyksvlei	7.59 *	1.28 *	1.51 *	0.96 *	7.63 *	-0.31 *
Vryburg	-0.02	0.54	0.28	-0.31	0.79	0.40
Mafikeng	-1.93	0.09	-0.39	-0.25 *	-1.48	0.83
*Average trend (95% CI)*	*3.29 (1.80-4.78)*	*0.57 (0.29-0.85)*	*0.32 (-0.01-0.65)*	*0.46 (0.15-0.77)*	*2.06 (0.50-3.61)*	*-0.21 (-0.59-0.17*)
*% stations with significant trends*	*50.0*	*35.7*	*28.6*	*35.7*	*28.6*	*21.4*

* significant at p < 0.05

Data presented include trend estimates for each weather station, trend estimates averaged across weather stations, and the % of weather stations displaying significant trends.

**Table 4:** Trend estimates for heat waves relevant to southern pied babblers (T_thresh_ = 35.5).

Weather station	# days > T_thresh_ (days/decade)	Heat wave frequency (days/decade)	Max. duration of heat waves (days/decade)	Av. intensity of heat waves (degree days/decade)	Max. intensity of heat waves (degree days/decade)	Av. interval between heat waves (days/decade)
Calvinia	1.76 *	0.90 *	0.20	0.12	0.75	-0.96 *
De Aar	-0.30	-0.17	-0.18	-0.16	-0.52	0.21
Fraserburg	1.49 *	1.07 *	0.17	0.21 *	0.85 *	-1.53
Henkries	3.40 *	0.60	1.22 *	0.01	5.95 *	-0.08
Kimberley	1.90	0.61	0.25	0.16	0.98	-1.30
Pofadder	3.13 *	1.18 *	0.43 *	0.20	1.35	-0.93 *
Prieska	1.76	0.68	0.39	0.04	1.37	-0.05
Sutherland	0.11 *	-	0.11 *	-	0.10 *	-
Twee Rivieren	7.81 *	0.43	1.28 *	1.03 *	6.34 *	-0.21
Upington	5.86 *	1.56 *	0.26	0.36	2.30	-0.25
Vaalharts	1.86	0.61	0.35	0.06	0.85	-0.03
Vanwyksvlei	5.48 *	1.80 *	0.42	0.36 *	1.97 *	-0.62 *
Vryburg	-0.38	-0.13	0.14	0.15	0.41	0.91
Mafikeng	-0.59	-0.26	-0.16	-0.08	-0.29	1.64
*Average trend (95% CI)*	*2.38 (1.06-3.70)*	*0.68 (0.34-1.03)*	*0.35 (0.13-0.57)*	*0.19 (0.03-0.35)*	*1.60 (0.52-2.68)*	*-0.25 (-0.72-0.22*)
*% stations with significant trends*	*57.1*	*35.7*	*28.6*	*21.4*	*35.7*	*21.4*

* significant at p < 0.05

Data presented include trend estimates for each weather station, trend estimates averaged across weather stations, and the % of weather stations displaying significant trends.

### Geographical patterns

**Figure 4:**
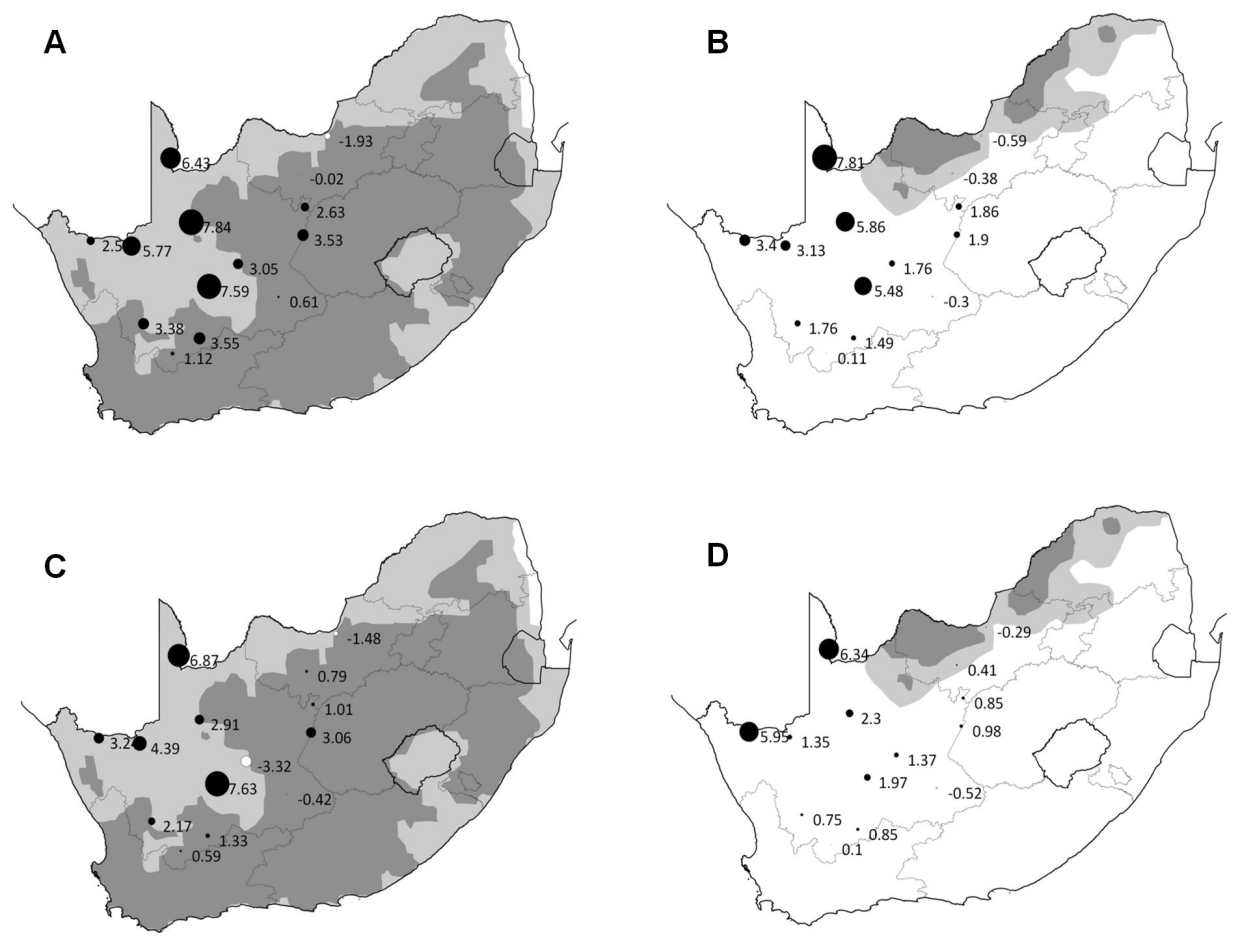
Decadal trends in the number of days T_max_ > T_thresh_, and in maximum heat wave intensity. Decadal trends in: the number of days exceeding T_thresh_ for A: fiscals and B: babblers; and the maximum intensity of heat waves for C: fiscals and D: babblers (degree days). The strongest trends for both indices occurred in the northwest of the study area. Circles denote weather station locations, labelled with the appropriate trend estimate. Circle size is proportional to the strength of the trend (effect size), black circles indicate positive trends, and white circles indicate negative trends. Fiscal distribution is shown in light and dark grey on maps A and C; babbler distribution is shown in light and dark grey on maps B and D [29]. Light grey regions: SABAP1 reporting rates < 70%; dark grey regions: SABAP1 reporting rates > 70% (see text for explanation).

Strongest warming trends in the number of days T_max_ > T_thresh_, the frequency of heat waves and the average and maximum heat wave intensity, were all found in northwest of the study area: i.e. the western Karoo and the southern Kalahari proper (examples given in Figure 4). Maximum duration of heat waves showed small, significant increases also in the western Karoo (fiscals) and southern Kalahari (babblers); in the order of 0.1 to 1.5 days/decade. Weather stations in the Northwest Province did not record warming trends, and in one case even recorded a significant cooling trend. Tables 3 (fiscals) and 4 (babblers) provide trend estimates for each weather station. 

## Discussion

Between 1961 and 2010, the frequency, intensity and maximum duration of biologically meaningful heat waves; regardless of whether defined with reference to babbler or to fiscal T_thresh_ values; all increased significantly within the southern Kalahari, Upper Karoo and Bushmanland bioregions of South Africa (trend estimates averaged across all stations). In addition, we recorded significant increases in the number of hot days (T_max_ > T_thresh_) per decade across the study region. However, evidence for a concurrent decrease in the intervals between heat waves was more equivocal. This trend may have been weakened by increases in annual frequency of heat waves (likely due to increasing length of the ‘hot’ season), resulting in a larger number of intervals between heat waves that averaged out to roughly the same length. The most rapid increases we recorded were in the number of hot days per decade and maximum heat wave intensity. The stronger warming trend in maximum, as opposed to average, heat wave intensity suggests that the variance in heat wave intensity increased along with the mean during this time period. If these trends continue, we should therefore expect greater uncertainty in the intensity of heat waves, and greater potential for intensity extremes of the type that have resulted in mass mortalities of birds and bats in Australia [5,7]. The most rapid and significant increases in all indices occurred in the northwest of the study area, with the weather stations at Twee Rivieren, Upington, Pofadder and Vanwyksvlei consistently recording strong trends.

### Implications of calculating heat wave trends using *T*_*thresh*_ values

**Figure 5:**
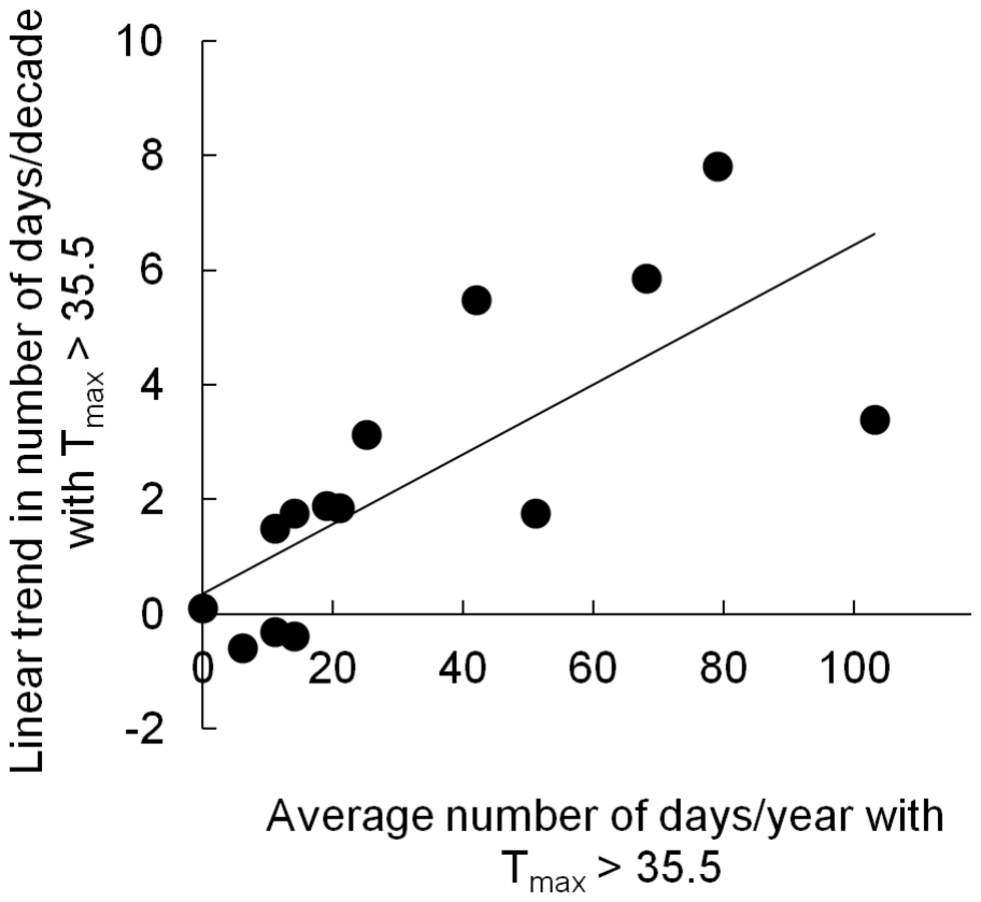
Rates of warming are highest in already-warm areas. Relationship between linear trend of days with T_max_ > 35.5°C and average number of days with T_max_ > 35.5°C, showing the tendency for areas that already experience large numbers of hot days (defined as T_max_ > T_thresh_) each summer to show more rapid gains in additional hot days under a regime of climate warming than cooler areas. Each data point represents a single weather station used in this study.

From a purely climatological perspective this study is largely consistent with the results from previous studies (e.g. 17), showing that the northwestern region of South Africa underwent probably the strongest warming in the country during the last five decades. In general, ‘hot’ weather stations that recorded high numbers of days annually on which T_max_ > T_thresh_ between 1961 and 2010, showed stronger decadal warming trends than ‘cool’ weather stations. For example, Figure 5 presents the trend estimates for the average number of days / decade with T_max_ > 35.5°C (babbler T_thresh_), plotted against the annual average number of days on which T_max_ > 35.5°C. The strong positive relationship apparent in this graph is likely due to the sine-wave shape of yearly temperature traces, meaning hot regions will, as a matter of course, appear to be warming faster than cool regions when trend analyses are based on absolute thresholds (e.g. T_thresh_ values). From a climatological viewpoint, therefore, such analyses produce less-meaningful results for climate change assessments, than those based on the historical distribution of temperatures, e.g. number of days above the 90^th^ percentile of a long-term historical base period; as such an index takes into account the inherent climatological properties of the measuring location [21]. From a biological viewpoint, however, each day above a biologically meaningful T_thresh_ is important in terms of its impact on fitness; regardless of the historical pattern of such days in the area. Birds in hot areas may therefore experience stronger effects of warming trends generally, as the physiological responses of endotherms to temperature are not linear, but invariably contain thresholds above which costs of thermoregulation rapidly increase [6,22,23].

### Biological implications of trends

Although all heat wave indices we investigated (with the exception of intervals between heat waves) showed warming trends, by far the most rapid increases we identified were in the numbers of days T_max_ > T_thresh_ (presumably the trend underlying the other increases we observed) and in the maximum intensity of heat waves. Variance in heat wave intensity also increased during the study period (as rates of change in maximum intensity outstripped average intensity), particularly in already-hot areas such as Twee Rivieren and Vanwyksvlei. These trends entail greater uncertainty in heat wave intensity and potential for intensity extremes. This may have significant biological implications, should these trends continue. 

High intensity heat waves can be lethal for endotherms (like birds) if temperatures overwhelm their capacity for physiological thermoregulation. This can happen because the metabolic costs of thermoregulation increase rapidly above upper critical temperature limits [23]. Furthermore, the costs of dissipating heat via evaporative water loss increase exponentially when ambient temperature exceeds body temperature for small birds [24]. McKechnie and Wolf [6] estimate that a small bird (~25 g) can survive for only 5.5 hours without access to water at 45°C air temperature. Even with access to replacement water, high intensity heat waves may still cause mortality if evaporative water loss is insufficient to counteract heat gain from the environment (for possible examples, see 5,25). When temperatures remain below lethal intensity, increases in the frequency and heat intensity of days on which T_max_ > T_thresh_ can still have severe consequences for individuals and populations. For example, in *Scleroporus* lizards in Mexico, high temperatures force the animals to cease activity and take refuge in rock crevices. Increasingly high T_max_ days correspond to an increased length of time per day when temperatures are above T_thresh_ for the lizards, reducing the time available for foraging and breeding activity and eventually resulting in population declines and extinctions [14].

The two studies from which we drew our estimates of babbler and fiscal T_thresh_ values [10,15]; provide some indication of how these species are likely to respond to high intensity heat waves. The costs of T_max_ exceeding babbler T_thresh_ (35.5°C) are manifest in terms of the inability of the birds to recoup body mass lost overnight. Du Plessis et al. [10] found that the differences between body mass gained during the day and body mass typically lost overnight increased in a linear fashion as T_max_ increased above T_thresh_, at least for T_max_ < 41°C (no data was collected at T_max_ > 41°C). Babblers began losing body mass during the course of the day when T_max_ > ~39.5°C. The authors propose that increased duration of heat waves may affect body mass in babblers in a cumulative manner, with implications for survival and breeding success. Increased intensity of heat waves seems likely to have a similar effect. However, the long-term effects of increased duration or increased intensity of heat waves, or, alternatively, extremely intense heat waves, are currently unknown for this species. Furthermore, the period of time babblers require to ‘recover’ from a hot weather event is also unknown. 

Fiscal T_thresh_ (33°C) was estimated on the basis of the negative impacts of T_max_ > T_thresh_ during the nestling period on fledgling mass in this species [15]. Reduced body mass at fledging often correlates with reduced survival probability and future breeding success in birds (reviewed by [26]). For fiscals, the impacts of a single day with T_max_ > T_thresh_ during the nestling period on fledging body mass depended on the degree to which T_thresh_ was exceeded. For example, each day over 33°C reduced fledging body mass by an average of 0.7 ± 0.4 g, whereas each day over 36°C reduced body mass by 1.3 ± 0.3 g, and each day over 38°C reduced body mass by 5.3 ± 1.8 g (data are means ± 1se, [15]). The effect of high T_max_ days therefore seems to increase in an accelerating, rather than linear, manner as the intensity of heat increases. In addition, both the number of days during the nestling period on which T_max_ > T_thresh_, and the timing of those days with respect to the stage of growth of the nestlings, appear to be important in determining the outcomes for fledging mass. However, no data exists for T_max_ > 38°C, and, as with the babblers, the long term fitness implications of the observed effects have not been measured. 

### Geographical patterns

The strongest and most significant increases in all heat wave indices, for both babblers and fiscals, occurred in the northwest of the study area. The weather stations at Twee Rivieren, Upington, Pofadder and Vanwyksvlei in particular consistently recorded strong trends, especially in the number of hot days and maximum heat wave intensity. By contrast, the weather stations at Mafikeng and Vryburg in the northeast of the study area recorded very small, in some cases negative, trends in all heat wave indices. This conforms to the findings of other climate change analyses for the region [17]. 

The rapidly-warming region we identified around Twee Rivieren, Upington, Pofadder and Vanwyksvlei is outside of the South African range of the southern pied babbler. Babblers are ‘thornbush specialists’ reliant on tall woodland stands for nesting sites [27,28] and their absence from the southwestern corner of the Kalahari basin, including the area mentioned above, is likely due to the lack of sufficient habitat there. The region around Mafikeng and Vryburg, however, does fall within the babbler’s range. If temperatures remain stable in this area into the future (as they did between 1961 and 2010), while temperatures in the core of the babbler’s range in Botswana increase (as predicted by [18]) this area could potentially become a climate refuge for the species, and may deserve protection as such.

Common fiscals, unlike babblers, are found throughout our study area. They are a habitat generalist, common across South Africa but largely absent from the central Kalahari basin in Botswana [20]. Reporting rates of fiscals (% of repeated surveys that recorded fiscals in the area: a proxy for population density) in the first Southern African Bird Atlas (SABAP1, [29]) 

are lowest in the arid periphery of the Kalahari and along the Namibian coast. Intriguingly, reporting rates are also relatively low in the region from Twee Rivieren to Vanwyksvlei compared to elsewhere in South Africa (see Figure 4). It was in this region that we recorded the most rapid warming trends in heat wave indices as defined by fiscal T_thresh_. This raises the tantalizing possibility that fiscal populations in this area may have already been affected by increased temperatures; and that, if trends continue and the birds are unable to adapt, the population in this area may become increasingly vulnerable. However, fiscals are widespread and loss from parts of the southern Kalahari and Bushmanland is unlikely to cause the species to become at risk of extinction within South Africa as a whole. More concerning, perhaps, is the potential, unknowable, impact on Kalahari ecosystems of losses of ‘peripheral’ species with low T_thresh_ values, like fiscals. 

### Analysing trends in other climate parameters

We have focused on analysing warming trends relevant to the fitness of the two study species. However, temperature is not the only climate variable showing changing trends under climate change. One variable that potentially has enormous biological significance is rainfall. The two papers from which we drew our T_thresh_s did not contain any information regarding potential rainfall thresholds (R_thresh_s) affecting aspects of the life-history of the focal species. Furthermore, it is uncertain whether species are likely to respond physiologically and behaviourally to rainfall patterns in the same manner as they respond to temperature. However, if it can be shown for any species that such a rainfall threshold exists (or for that matter, thresholds in any other climate variable, or combination thereof, for which long term data sets are available), then our threshold technique could be applied to these variables as well.

### Implications for conservation

The T_thresh_ approach facilitates examination of warming trends relevant to any fitness correlate for which data exists from the species of interest. In our examples, exceeding T_thresh_s affected the ability of adult babblers to maintain body mass, and caused fiscals to fledge from the nest at a smaller body mass [10,15]. The result for fiscals is important, because they breed in response to rainfall, as do many arid-zone birds [30]. They are therefore not likely to be able to shift breeding dates earlier to avoid increasingly hot summer temperatures, as some temperate species have been documented to do, e.g. [31,32].

In some instances, data might be available for more than one temperature threshold (e.g. a number of known T_thresh_s exist, relevant to different aspects of the life history of a species). Managers can choose which T_thresh_ to use based on the magnitude of associated fitness costs, or based on which T_thresh_ is lowest. Under some circumstances, population modelling may also be of use in deciding among multiple T_thresh_s: the T_thresh_ related to the life-history stage identified as having most impact on population trajectories can then be chosen. Even in cases where data is sparse and only a single T_thresh_ is known (as in the babbler example), analysis of warming trends with respect to this T_thresh_ will still provide more biologically meaningful information than standard climate analyses. This is because we can be confident that any heat wave defined using a T_thresh_ above which known fitness costs are incurred will affect the fitness of the focal species.

In our study, applying the T_thresh_ technique to analyses of past climatic trends allowed us to ascertain that the frequency, duration and intensity of hot weather events, *as relevant to our two focal species*, all increased between 1961 and 2010 in our study area. Furthermore, we were able to show that maximum heat wave intensity had increased faster than other indices, including average intensity. This suggested that increased variance in intensity, with the corollary of increased likelihood of intensity extremes, was the aspect of warming most likely to have already affected our study species. Potentially in support of this, SABAP1 reporting rates for fiscals in the most rapidly-warming region of our study area were lower than in other areas of their range which experienced weaker warming trends [20]. Should the trends we identified in this study continue into the future, then the T_thresh_ technique predicts we should focus our research efforts on the form of each species’ response to high intensity heat waves, and the longer term implications for these for the life-history of individuals.

As illustrated by southern pied babblers and common fiscals, the T_thresh_ method allows identification of the heat wave trends with the most immediate likelihood to impact focal species of interest, and geographical patterns in these trends. We chose to investigate trends within a single region, but this approach could be applied across entire species ranges, provided data is available, allowing both areas of greatest risk and potential climate refugia to be identified. For species that are already flagged as at risk due to climate change induced warming (e.g. the Ethiopian bush-crow *Zavattariornis stresemanni*, [33]; or European birds with low temperature tolerances, [34]), the application of this approach could guide both the collection of data on species’ responses to appropriate aspects of temperature rise, and also aid significantly in conservation planning. 
